# Sodium MRI with 3D-cones as a measure of tumour cellularity in high grade serous ovarian cancer

**DOI:** 10.1016/j.ejro.2019.04.001

**Published:** 2019-04-19

**Authors:** Surrin S. Deen, Frank Riemer, Mary A. McLean, Andrew B. Gill, Joshua D. Kaggie, James T. Grist, Robin Crawford, John Latimer, Peter Baldwin, Helena M. Earl, Christine A. Parkinson, Sarah A. Smith, Charlotte Hodgkin, Elizabeth Moore, Mercedes Jimenez-Linan, Cara R. Brodie, Helen C. Addley, Susan J. Freeman, Penelope L. Moyle, Evis Sala, Martin J. Graves, James D. Brenton, Ferdia A. Gallagher

**Affiliations:** a Department of Radiology, University of Cambridge, Cambridge, CB2 0QQ, United Kingdom; b Cambridge University Hospitals NHS Foundation Trust, Addenbrooke’s Hospital, Cambridge, CB2 0QQ, United Kingdom; c Cancer Research UK Cambridge Institute, University of Cambridge, Cambridge, CB2 0RE, United Kingdom

**Keywords:** Sodium MRI, Magnetic resonance imaging, Ovarian cancer, Tumour cellularity

## Abstract

The aim of this study was to assess the feasibility of rapid sodium MRI (^23^Na-MRI) for the imaging of peritoneal cancer deposits in high grade serous ovarian cancer (HGSOC) and to evaluate the relationship of ^23^Na-MRI with tumour cellularity. ^23^Na-MRI was performed at 3 T on twelve HGSOC patients using a 3D-cones acquisition technique. Tumour biopsies specimens were collected after imaging and cellularity was measured from histology. Total ^23^Na-MRI scan time for each patient was approximately 11 min. At an isotropic resolution of 5.6 mm, signal-to-noise ratios (SNRs) of 82.2 ± 15.3 and 15.1 ± 7.1 (mean ± standard deviation) were achieved for imaging of tumour tissue sodium concentration (TSC) and intracellular weighted sodium concentration (IWS) respectively. Tumour TSC and IWS concentrations were: 56.8 ± 19.1 mM and 30.8 ± 9.2 mM respectively and skeletal muscle TSC and IWS concentrations were 33.2 ± 16.3 mM and 20.5 ± 9.9 mM respectively. There were significant sodium concentration differences between cancer and skeletal muscle, Wilcoxon signed-rank test, *P* <  0.001 for TSC and *P* =  0.01 for IWS imaging. Tumour cellularity displayed a strong negative correlation with TSC, Spearman’s rho = -0.92, *P* <  0.001, but did not correlate with IWS. This study demonstrates that ^23^Na-MRI using 3D-cones can rapidly assess sodium concentration in peritoneal deposits of HGSOC and that TSC may serve as a biomarker of tumour cellularity.

## 1 Introduction

In the developed world, ovarian cancer is the fifth leading cause of cancer-related mortality in women and high grade serous ovarian cancer (HGSOC) is responsible for 70–80% of these deaths [1]. HGSOC originates in the secretory cells of the fallopian tubes and metastasises to the peritoneum and ovaries early on in the course of the disease [2]. The peritoneal deposits of HGSOC are typically superficially located and easily accessible to percutaneous biopsy. For this reason, peritoneal HGSOC tissue is often sampled for diagnostic purposes in preference to the deeper and less accessible ovarian deposits.

HGSOC lesions demonstrate significant intra- and intertumoural heterogeneity that can affect progression and survival [3]. Multi-regional sampling of tissue and repeat sampling during therapy however is often not practical due to the invasive nature of the biopsy procedure. Imaging provides an alternative to biopsy for investigating entire heterogeneous tumour volumes and for non-invasively evaluating cancer progress over time.

In human tissue the sodium ion concentration is approximately ten times higher in the extracellular compartment compared to the intracellular space. Intracellular sodium concentration [Na^+^_IC_] is usually around 5–15 mM and extracellular sodium concentration [Na^+^_EC_] ranges from 135 to 155 mM. Overall tissue sodium concentration (TSC) is a weighted average of the intracellular sodium concentration and the extracellular sodium concentration. The weighting in this average is related to the cellularity of the tissue as higher cellularity corresponds to a greater fraction of intracellular space and consequently a lower TSC. Measurements of TSC could therefore potentially be used to probe cellularity and changes in cellularity such as with cell death in response to chemotherapy.

The free movement of sodium between the intracellular and extracellular compartments is normally restricted by the phospholipid bilayer of the cell membrane which is poorly permeable to sodium. The majority of sodium ions that cross the cell membrane do so through specialized ion transporters like the Na^+^/K^+^-ATPase pump, also known as the sodium pump. Cell membrane porosity increases occurs early on in the process of cell death, allowing greater sodium movement into the cell down the electrical and concentration gradients that exist across the membrane. This intracellular influx of sodium is associated with an osmotic movement of water that contributes to cell swelling prior to death [4]. An increase in TSC has previously been shown to be an early marker of necrosis and part of the process of apoptosis [5,6]. Sodium MRI (^23^Na-MRI) can measure TSC and the ability of ^23^Na-MRI to assess tissue viability in the brain after acute stroke has already been demonstrated in several studies [7,8]. In murine models of glioma and prostate cancer, ^23^Na-MRI has also successfully detected the response of tumour to chemotherapy [9,10]. In HGSOC, the most effective therapeutic strategies combine surgery and chemotherapy [11]. If TSC measured using ^23^Na-MRI can detect cell viability and cellularity in HGSOC, then ^23^Na-MRI may be of clinical value for monitoring both the early and late tissue changes following successful chemotherapy treatment, as responding cells first undergo an increase in sodium and swelling before death, followed by a decrease in tumour cellularity and intracellular volume fraction as the process of death completes.

In ^23^Na-MRI, the intracellular weighted sodium (IWS) signal can also be separated from the overall tissue sodium signal using an inversion recovery pulse sequence that exploits a difference in the T_1_ relaxation times of free and bound sodium. Increased TSC and IWS concentrations compared to normal tissue have been found using ^23^Na-MRI in several cancers including brain, breast and prostate [[12], [13], [14]]. In the abdomen however, ^23^Na-MRI has proved technically challenging. ^23^Na-MRI generates a signal which is 22,000-fold smaller than conventional proton MRI (^1^H-MRI) [15] and the sodium nucleus rapidly loses signal after radiofrequency (RF) excitation because of a high sensitivity to electrical fields.

Longer scan times that permit signal averaging can compensate for the low ^23^Na-MRI signal. Previous studies of the abdomen have reported scan times of 16–25 min for the generation of quantitative sodium maps [[16], [17], [18]]. Such long scan times unfortunately may not always be feasible in clinical settings. A 3D-cones trajectory approach to ^23^Na-MRI in the brain however was recently shown to improve acquisition times compared to the more widely used 3D-radial *k*-space sampling methods, without any compromise in image quality [19]. Here we investigate the application of ^23^Na-MRI using 3D-cones to the imaging of peritoneal cancer deposits in HGSOC and explore the relationship of histologically measured tumour cellularity with the imaging.

## 2 Materials and methods

### 2.1 Study conduct

This was a single centre prospective observational study (ClinicalTrials.gov Identifier: NCT03526809) with institutional review board (IBR) approval (South Cambridge Research Ethics Committee reference number 15/EE/0378). All study related procedures were performed with the written informed consent of participants and in accordance with the ethical guidelines outlined in the Declaration of Helsinki. Consecutive HGSOC patients presenting from August 2016 to August 2017 with no contraindications to MRI and no previous treatment for their cancer were invited to participate.

### 2.2 Image acquisition

^23^Na-MRI and ^1^H-MRI were carried out on all patients using a 3 T MR system (MR750 GE Healthcare, Waukesha WI). The ^23^Na-MRI was performed with a custom made single-channel transmit/receive surface coil using a 3D-cones readout [20] and an adiabatic pulse for inversion at a prescribed isotropic resolution of 5.6 mm. T_2_-weighted ^1^H-MRI was performed with a fast spin echo pulse sequence and a 32-channel cardiac array. Patients were not moved or repositioned during the coil change. The radiofrequency (RF) power used for the ^23^Na-MRI was adjusted for each patient to achieve penetration up to a maximum depth of 12 cm which was sufficient to image the peritoneal lesions in all cases. Detailed scan parameters for sodium and proton imaging are given in Table 1.

**Table 1:** Imaging parameters for TSC (total sodium concentration), IWS (intracellular weighted sodium) and T_2_-weighted imaging. TR = repetition time, TE = echo time, TI = inversion time, FoV = field of view, NEX = number of excitations, ETL = echo train length, GE = gradient echo.

Imaging parameter	^23^Na-MRI TSC and B_1_ mapping	^23^Na-MRI IWS	^1^H T_2_-weighted
TR	100 ms	250 ms	4000 ms
TE	0.5 ms	0.5 ms	91.1 ms
TI	n/a	30 ms	n/a
Flip angle	90° for TSC, 30° and 60° for B_1_ mapping	90°	90°
Slice thickness	5.6 mm	5.6 mm	6 mm
In plane resolution	5.6mmx5.6mm	5.6 mm x 5.6 mm	1.33 mm x 1.33 mm
FoV	30 cm	30cm	34.0 cm x 29.9 cm
Matrix	50 × 50 (reconstructed to 120 × 120)	50 × 50 (reconstructed to 120 × 120)	256 × 256
NEX	6	6	8
ETL	n/a	n/a	16
Total scan time	1 min 58 sec	4 min 56 sec	1 min 54 sec
Pulse sequence	GE with 3D-Cones readout	IR- GE with 3D-Cones readout	Single shot fast spin echo (SSFSE)

Sodium calibration phantoms were included in the field of view (FoV) to enable calculation of sodium concentration maps. Phantoms consisted of 50 mL tubes of diameter 6 cm containing sodium chloride (NaCl) with 4% agar at two sodium concentrations: 20 mM and 80 mM.

Image processing and noise correction

Sodium image reconstruction and post-processing were performed with in-house software written in MATLAB v9.2 (The MathWorks Inc., Natick, MA). The method described by Miller et al. was applied for noise correction [21]: this correction involved squaring the signal derived from the image, measuring the squared mean pixel value from a region of interest (ROI) in the background (outside the patient) for each slice, subtracting this value from all pixels in the squared image and calculating the square root. All signal-to-noise ratios (SNRs) reported in the results section of this study were calculated prior to noise correction as the mean signal divided by the standard deviation of background signal [22].

Radiofrequency field inhomogeneity correction

To compensate for RF inhomogeneity in the FoV, a B_1_ correction was applied to the sodium images. A dual flip angle gradient echo method [23] with images collected at nominal flip angles ($\alpha_{nom}$) of 30° and 60° was used for the B_1_ mapping to create an RF map (B_1_ map) of the true flip angle $\alpha_{true}$. Regions on the B_1_ maps which had very low effective flip angles (<10°) or high flip angles (>140°) where dual angle mapping is inaccurate [24] were excluded from subsequent analysis.

A correction formula was derived to use $\alpha_{true}$ to compensate for the non-uniformities in both the transmit and the receive fields. To do this, the expression for the spoiled gradient echo (GE) steady state signal was combined with the relationship of signal to the receive-only B_1_ of a surface coil [25]. For the sodium pulse sequence used here TR >> T_1_, causing the overall relationship of the combined receive and transmit signal to reduce to approximately:

(1) $$\left[{Na}^{+}\right]\propto \frac{S}{\alpha_{true}.sin(\alpha_{true})}$$

Where S is the sodium image signal. Eq. (1) was applied to correct the sodium signal images before conversion into sodium concentration maps.

### 2.3 Region-of-interest analysis

Sodium maps (TSC and IWS) were calculated from a linear calibration curve created with the sodium in agar phantoms based on the method described by Christensen JD et al. [26]. ROIs were drawn on the T_2_-weighted images by a single observer using OsiriX (version 3.8.1, Pixmeo, Geneva, Switzerland) around the complete peritoneal deposits and around the adjacent areas of right gluteal muscle as a normal reference tissue for comparison with the tumour. ROIs were reviewed by a radiologist with eight years of experience as an attending physician and co-registered with the sodium maps to which the ROIs were imported for analysis. For patients with multiple peritoneal deposits, ROIs were combined into a single tumour volume.

### 2.4 Tissue handling

Tissue samples were collected from the peritoneal deposits of patients 1–14 days (median 7 days) after imaging either by ultrasound guided biopsy or at surgery. Samples were fixed in paraffin blocks for storage and cut into 3 μm sections that were retrieved with sodium citrate. Sections were stained with haematoxylin and a cell count and tissue area quantification were performed on the sections using automated histology image analysis software (Halo, v2.1.1637.11, Indica labs). Cellularity (cell density), $\rho_{c}$ was estimated from the cell count (N) and tissue area (A) in μm^2^ as follows:

(2) $$\rho_{c}=\;N\;/\;A\;x\;1000$$

### 2.5 Statistics

All statistical analysis was performed in R (v2.15.3, R Foundation for Statistical Computing, Vienna, Austria). For comparison of means, the Shapiro-Wilk test was used to assess data for normality and the Student’s *t*-test or Wilcoxon test was then applied to evaluate significance. Cellularity was compared to sodium concentrations from ^23^Na-MRI (TSC and IWS) using Spearman’s correlation.

## 3 Results

### 3.1 Patients

Twelve high grade serous ovarian cancer patients were recruited: median age 69 (range 52–81) years. Table 2 gives detailed sample population characteristics.

**Table 2:** Population demographics of patients recruited. ECOG = Eastern Cooperative Oncology Group, FIGO = Fédération Internationale de Gynécologie et d'Obstétrique, CA 125 = cancer antigen 125.

Feature	Value
Number of patients	12
Age, median (range, years)	69 (52-81)
ECOG performance status (number of patients)	
0–2	9
3–4	3
FIGO stage (number of patients)	
I	0
II	2
III	8
IV	2
Serum CA 125 (IU/ml, number of patients)	
0–100	4
100–500	3
>500	5

### 3.2 Imaging

**Fig. 1:**
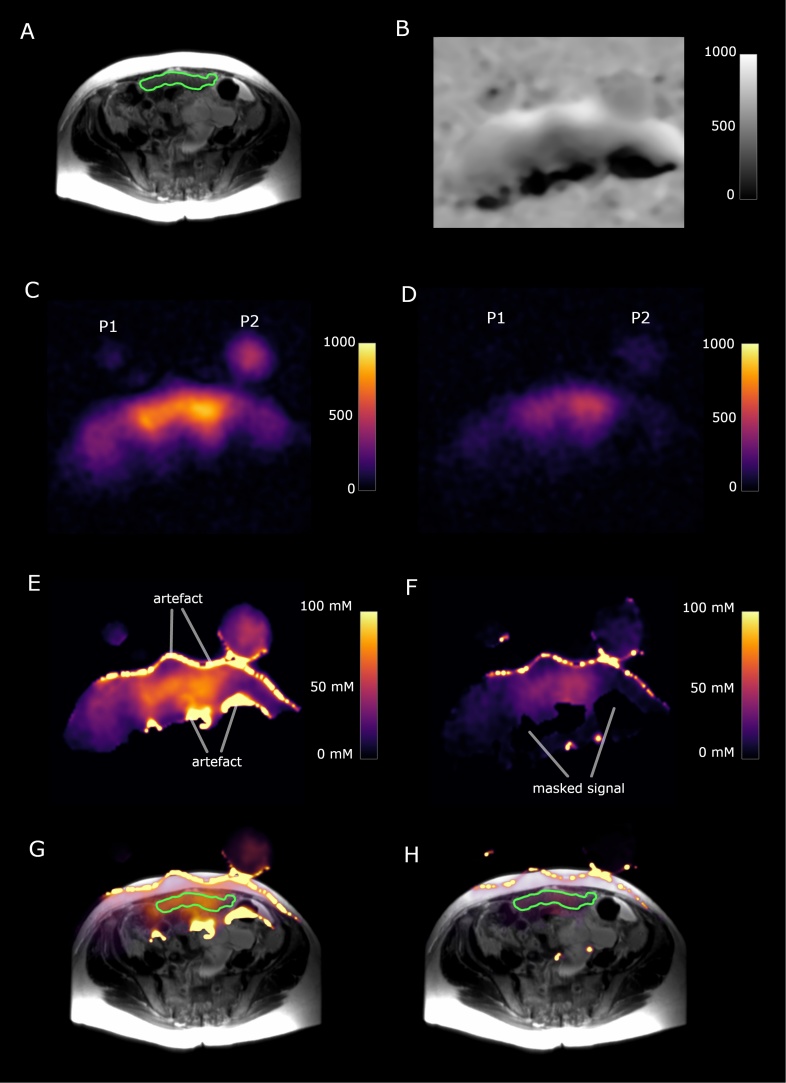
73-year old high grade serous ovarian cancer patient. P1 and P2 represent slices through the two sodium phantoms. The green outline shows a peritoneal cancer deposit. (A) T_2_-weighted image. (B) Sodium B_1_ map, scale bar represents arbitrary units. (C) Total sodium image; scale bar represents image intensity. (D) Intracellular weighted sodium image; scale bar represents image intensity. (E) Masked total sodium concentration map; scale bar represents sodium concentration in mM. (F) Masked intracellular weighted sodium concentration map, scale bar represents sodium concentration in mM. (G) Fused T_2_W image and total sodium concentration map. (H) Fused T_2_W image and intracellular weighted sodium concentration map. (For interpretation of the references to colour in this figure legend, the reader is referred to the web version of this article).

The total ^23^Na-MRI imaging time for each patient was under 11 min (TSC imaging time = 1 min 58 s, IWS imaging time = 4 min. 56 s, time for images for dual flip angle mapping = 3 min 56 s). Signal-to-noise ratios of 82.2 ± 15.3 and 15.1 ± 7.1 were achieved for tumour TSC and IWS imaging respectively. High signal intensity artefacts appeared at the edges of images after RF field inhomogeneity corrections because of small mismatches in alignment between B_1_ maps and sodium signal images at the interfaces of high sodium signal changes such as between air and tissue. These mismatches were due to patient movement and were found exclusively at imaging borders and therefore were not present in ROIs derived from peritoneal deposits or muscle. Examples of the ^23^Na-MRI images and changes with post-processing are shown in Fig. 1.

### 3.3 Sodium quantification and tumour cellularity

**Fig. 2:**
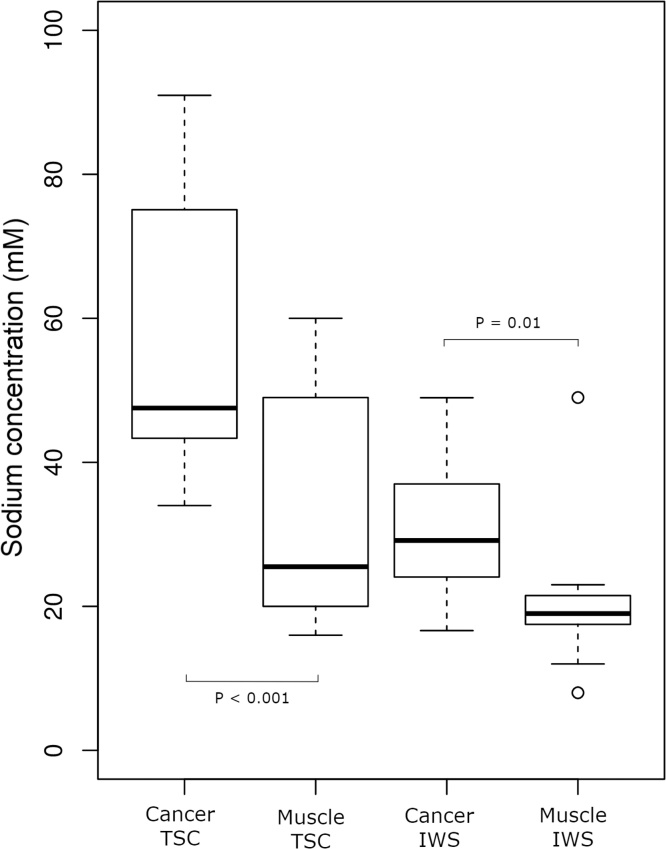
Box plots showing the spread of TSC (total sodium concentration) and IWS (intracellular weighted sodium) values for cancer and muscle.

The TSC for peritoneal cancer deposits and gluteal skeletal muscle were 56.8 ± 19.1 mM and 33.2 ± 16.3 mM respectively (mean ± standard deviation); Wilcoxon signed-rank test, *P* <  0.001. IWS values for peritoneal cancer and muscle were 30.8 ± 9.2 mM and 20.5 ± 9.9 mM respectively; Wilcoxon signed-rank test, *P* =  0.01. These results are summarised in the boxplots of medians and interquartile ranges shown in Fig. 2.

**Fig. 3:**
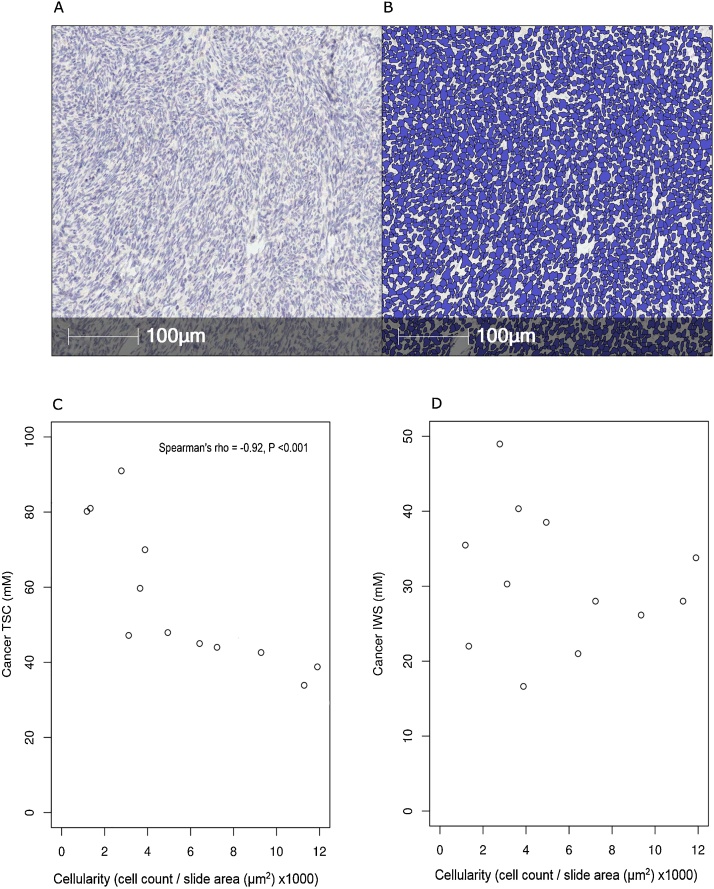
Tumour tissue from a 73-year old high grade serous ovarian cancer patient; (A) 1x magnification haematoxylin stained slide of tumour tissue; (B) automated cell segmentation used for cell counting. Scatterplots of; (C) tissue sodium concentration against cellularity, (D) intracellular weighted sodium concentration against cellularity.

An example of the typical histological appearances for a 73-year old HGSOC patient are shown in Fig. 3 together with the automated cell counting segmentation, demonstrating the accuracy of the software at detecting cells. Scatterplots of tumour cellularity ($\rho_{c}$) compared with sodium concentrations as measured on imaging are shown in Fig. 3(C) and 3(D). TSC displayed a strong negative correlation with cellularity, Spearman’s rho = -0.92 and *P* <  0.001. There was no significant correlation found between IWS concentration and cellularity, *P* =  0.44.

## 4 Discussion

This study demonstrated the feasibility of using ^23^Na-MRI to image peritoneal lesions in high grade serous ovarian cancer patients for the first time. The completion of sodium imaging in approximately eleven minutes was achieved with a combination of a 3D-cones readout, a surface transmit/receive coil and a post-processing analysis method to correct for RF field non-uniformity. Short acquisition times are important for the clinical translation of ^23^Na-MRI and these results represent a moderate improvement to previously reported scan times in the abdomen [[16], [17], [18]]. The comparison of tumour sodium quantification to histology that was performed here, also provided validation that TSC measured by ^23^Na-MRI is an effective biomarker for tumour cellularity.

Cellularity has already been shown to relate to the outcome of cytoreduction in HGSOC [27] and to be predictive of the response of breast cancer to neoadjuvant chemotherapy [28]. In breast cancer, cellularity is also used more directly in the Miller-Payne method for the histological assessment of treatment response [29]. For HGSOC treatment, the first line chemotherapy regimen of a combination of a platinum-based drug and a taxane has not changed for the past 30 years despite poor patient outcomes [30]. There is now ongoing research to develop more targeted HGSOC therapies such as PARP (poly ADP-ribose polymerase) inhibitors [31], VEGF (vascular endothelial growth factor) inhibitors [32] and immune checkpoint inhibitors [33], to improve the outlook for this disease.

With the emergence of new therapies in heterogeneous cancers like HGSOC, there is an associated need for imaging to provide greater detail on tumour structure and function so that patients can be better stratified to the optimal treatment for their particular subtype of disease. The availability of alternative therapies also provides the opportunity for non-responsive patients to switch to more effective drugs and imaging tests that can assess response are therefore similarly required. The measurement of cellularity with ^23^Na-MRI demonstrated here provides information on tumour composition and can be employed to monitor the evolution of tumour cellularity over time and following treatment. This could additionally have implications for patient management in other malignancies where the detection of cell death and the monitoring of cellularity changes are of clinical relevance like in breast cancer.

Apart from cellularity, ^23^Na-MRI also reflects the activity of transporters such as the Na^+^/K^+^-ATPase pump, and possibly by extension ATP levels, which may be altered earlier than cellularity in some physiological processes like cell death. ^23^Na-MRI could therefore potentially report on response to treatment earlier than other imaging methods like diffusion weighted MRI, which primarily also detects cellularity and probe processes like metabolism and transporter activity which diffusion cannot.

Despite the strong significance of the relationship between TSC and cellularity found here, no correlation between cellularity and IWS could be detected. IWS concentration in cancer is influenced by a large number of biological processes that may have a greater relative effect on the small IWS concentration and imaging signal than cellularity. For example, the IWS signal may be affected by the energy status of tumours due to the ATP and oxygen requirements of the sodium pump [34]. In cancer, there may be mitochondrial dysfunction [35] as well as metabolic reprogramming that shifts glucose away from the production of ATP by oxidative phosphorylation and towards the manufacture of new nucleotides, lipids and amino acids needed for cell replication and unregulated tumour growth [36,37]. Voltage gated sodium channel expression is also upregulated in many cancers [38,39] and although the purpose of these channels in cancer is not yet clear, they may represent another mechanism by which the intracellular sodium concentration is altered in malignancy. Furthermore, aggressive tumours such as HGSOC have higher Na^+^/H^+^ antiporter activity which allows sodium to enter the cell in exchange for H^+^ efflux, and creates an acidic extracellular environment that facilitates tumour invasion [40].

TSC signal is dominated by the high concentration of sodium in the extracellular space and is less susceptible to the transmembrane movement of sodium as it is a measure of the total tissue sodium in each voxel, including signal from sodium in both the intracellular and extracellular environments. By comparison, the IWS pulse sequence nulls the large extracellular sodium pool with its inversion recovery, resulting in a much lower SNR and greater susceptibility of IWS measurements to noise.

The conclusions that can be drawn from the findings in this study are limited by the possibility of errors introduced through patient movement during the sodium/proton coil change and the creation of edge artefacts in post-processing. The optimisation of RF power to image superficial peritoneal lesions also meant that muscle sodium measurements could not include deeper muscle regions. Reasonable measures were taken to minimise the effects of these limitations by masking possible inaccurate sodium map areas and the application of an RF inhomogeneity correction.

## 5 Conclusion

In conclusion, rapid ^23^Na-MRI using 3D-cones was successfully performed in a technically challenging area of the body. This study shows for the first time that ^23^Na-MRI can assess peritoneal deposits of HGSOC patients and that the TSC measured by ^23^Na-MRI correlates strongly with tumour cellularity. The non-invasive quantification of sodium using MRI has the potential to provide information on cell viability, sodium transporters, metabolic activity, cell membrane integrity and tumour response to therapy. The rapid scanning method shown here demonstrates that larger human studies to evaluate ^23^Na-MRI in the abdomen are feasible and should be supported. Further work is needed to investigate other clinical applications of sodium MRI, to explore the utility of TSC in monitoring cellularity changes in response to specific cancer treatments and to validate the cellular changes that can be measured with ^23^Na-MRI.

## Role of the funding source

To provide funding for the costs of this research. Funding was obtained from a Cancer Research UK grant (Grant reference C19212/A16628).

## Conflict of interest statement

The authors have no conflicts of interest to declare.
